# LC-HRMS fingerprinting and chemometrics for the characterization and classification of *Lotus* cultivars from Uruguay: a study on phenolic composition

**DOI:** 10.3389/fmolb.2025.1646758

**Published:** 2025-08-29

**Authors:** Cristina Olivaro, Nerea Núñez, Patricia Basile, América Mederos, Rafael Reyno, Javier Saurina, Oscar Núñez

**Affiliations:** ^1^ Espacio de Ciencia y Tecnología Química, CENUR Noreste, Universidad de la República, Tacuarembó, Uruguay; ^2^ Department of Chemical Engineering and Analytical Chemistry, University of Barcelona, Barcelona, Spain; ^3^ Espacio de Biología Vegetal del Noreste, CENUR Noreste, Universidad de la República, Tacuarembó, Uruguay; ^4^ Sistema Ganadero Extensivo, Instituto Nacional de Investigación Agropecuaria, Tacuarembó, Uruguay; ^5^ Mejoramiento Genético y Biotecnología Vegetal y Área de Pasturas y Forrajes, Instituto Nacional de Investigación Agropecuaria, Tacuarembó, Uruguay

**Keywords:** Lotus cultivars, classification, identification of phenolic compounds, UHPLC-HRMS/MS, non-targeted metabolomics, chemometrics

## Abstract

**Introduction:**

The *Lotus* genus, part of the legume family, comprises over 180 species distributed across diverse ecosystems worldwide. Its broad genetic diversity enables adaptation to various environmental conditions and represents a valuable resource for breeding programs targeting key agronomic traits. One of the most attractive features of *Lotus* species is the presence of condensed tannins in the forage, which, in ruminants, help prevent bloat, exhibit antiparasitic properties, enhance the absorption of non-ammonia nitrogen compounds, and reduce greenhouse gas emissions.

**Aims and methods:**

This study aimed to develop a UHPLC-HRMS method for classifying ten *Lotus* cultivars produced in Uruguay using a non-targeted metabolomic fingerprinting approach. Five cultivars belong to *Lotus corniculatus*, three to *Lotus uliginosus*, and two are interspecific hybrids. The analysis focused on phenolic compound-rich fingerprints. Principal component analysis (PCA) and partial least squares-discriminant analysis (PLS-DA) were used for data exploration and classification, and to identify key phenolic compounds with high discriminant potential. Finally, cultivar-specific polyphenolic compounds were tentatively identified based on chromatographic and high-resolution mass spectrometry (HRMS/MS) data obtained from all cultivars.

**Results:**

When defining four classes (*L. uliginosus*, *L. corniculatus*, and the two hybrids), the optimal PLS-DA model required six latent variables and achieved 100% classification accuracy, with both sensitivity and specificity reaching 100%. Additional PLS-DA models were developed to assess intra-species discrimination among the 3 *L. uliginosus* and 5 *L. corniculatus* cultivars, with varying degrees of separation observed. In each PLS-DA model, VIP loadings scores allowed the selection of the most discriminant phenolic compounds for each class under study. A total of 105 compounds, including phenolic acids, flavonols, flavan-3-ols, proanthocyanidins, and organic acids, were tentatively identified by analyzing all cultivars.

## 1 Introduction

The *Lotus* genus belongs to the legume family and comprises more than 180 species. The main regions of the world where *Lotus* species are cultivated are South America, North America, and Europe. In Uruguay, five *Lotus* species have been domesticated and improved through selection and breeding: *Lotus corniculatus* L., *Lotus uliginosus* Schkuhr., *Lotus tenuis* Waldst. & Kit. ex Willd., *Lotus angustissimus* Ledeb., and *Lotus subbiflorus* Lag. These species are mainly used either in mixtures with forage grasses or introduced into natural grasslands (Ayala and Carámbula, 2009). Considered bioactive forages due to their secondary metabolites, they can adapt to diverse soil types, tolerate acidity, and grow under low phosphorus conditions (Escaray et al., 2012).

Phenolic compounds are the most abundant and widely distributed secondary metabolites in plants. Structurally, phenolics consists of one or more hydroxyl groups attached to a six-carbon aromatic ring. These compounds range from simple phenolic acids to complex polymeric structures such as tannins. Over 8,000 phenolic structures have been identified to date, and they are commonly grouped into classes, including phenolic acids, flavonoids, xanthones, stilbenes, lignans, and coumarins, based on the number of phenolic rings and the linkages between them (Zhang et al., 2022). Phenolic acids contain a carboxylic acid functional group and are present in both free and bound forms. They are mainly classified into hydroxybenzoic acids (C6-C1) and hydroxycinnamic acids (C6-C3). Hydroxybenzoic acids are components of complex structures such as hydrolysable tannins (e.g., gallotannins and ellagitannins). Flavonoids constitute the largest and most structurally diverse class of phenolic compounds, consisting of a C6-C3-C6 carbon framework with specific hydroxylation patterns. They occur as free aglycones or, more frequently as O- and C-glycosides. Based on the oxidation state and saturation of the central heterocyclic ring, flavonoids are divided into subclasses, including flavones, isoflavones, flavanones, flavonols, dihydroflavonols, flavan-3-ols, flavan-4-ols, flavan-3,4-diols, anthocyanidins, and chalcones. Among these, flavan-3-ols can polymerize to form condensed tannins, also known as proanthocyanidins (PAs). PAs are classified as homo- or heteropolymers based on whether their constituent monomers are identical or different. They are further subclassified into types A and B, according to the nature of the carbon–carbon linkages between monomers. Compounds containing (epi)catechin are referred to as procyanidins, while those composed of (epi)gallocatechin or (epi)afzelechin are known as prodelphinidins or propelargonidins, respectively. Flavonols are among the most abundant monomeric flavonoids in plants, with quercetin, kaempferol and myricetin being the representative compounds. Supplementary Figure S1 in the Supplementary Material shows the structure of various phenolic compounds (Manach et al., 2004; Al Mamari, 2021).

Phenolic compounds play crucial roles in plant physiology, affecting responses to both biotic and abiotic stresses, and modulating interactions with the environment, including soil microbiota and pollinators. Beyond their functions in plants, these compounds' biological activities have garnered significant interest due to their potential applications in agriculture, animal nutrition, and human health. In farm animals, phenolic compounds exhibit antioxidant, anti-inflammatory, and anthelmintic properties, while also enhancing cell-mediated immunity and modulating ruminal and intestinal microbiota (Bhuyan and Basu, 2017; Mahfuz et al., 2021; Rahman et al., 2021; Serra et al., 2021; Pratyusha, 2022; Sun and Shahrajabian, 2023; Waqas et al., 2023).

In *Lotus* species, condensed tannins are particularly relevant, as they enhance protein utilization in ruminants, reduce methane and ammonia emissions, help prevent pasture bloat, exhibit antihelmintic activity, and improve animal productivity (Blumenthal et al., 1993; Molan et al., 2000; Mueller-Harvey, 2006; Waghorn, 2008; Jonker and Yu, 2017; Mueller-Harvey et al., 2019; Verma et al., 2022; Brutti et al., 2023). In addition, other polyphenols (such as flavonoids and phenolic acids) are highly effective, exerting strong positive effects on animal health and the quality of livestock products. They improve both the quantity and quality of meat and milk, reduce the need for synthetic antioxidants and antibiotics, and have a beneficial impact on the immune system (Luzardo et al., 2019; Serra et al., 2021; Formato et al., 2022; Waqas et al., 2023).

However, establishing direct correlations between phenolic structures and their biological functions remains a major challenge due to their chemical complexity and the limited understanding of their mechanisms of action. This challenge is further compounded by the potential interactions among the various compounds present in the cultivar, which may result in synergistic or antagonistic effects. Despite the agronomic and functional importance of *Lotus* species in sustainable grazing systems and soil improvement, the phenolic composition of many cultivars remains poorly characterized, since previous works have only been able to determine total phenolics, total tannins, or total flavonoids (Montossi, 1996; Reyno et al., 2022).

The main objectives of this study were to develop and apply a non-targeted ultra-high-performance liquid chromatography coupled to high-resolution mass spectrometry (UHPLC-HRMS) method, using a linear ion trap (LTQ)-Orbitrap analyzer, to classify and characterize ten *Lotus* cultivars produced in Uruguay, including four experimental lines. Chemical fingerprints were generated based on feature intensity across mass-to-charge (*m/z*) ratios and chromatographic retention times. These datasets were analyzed using principal component analysis (PCA) and partial least squares-discriminant analysis (PLS-DA) to assess cultivar classification and identify key phenolic markers with high discriminative potential. Finally, a tentative identification of characteristic polyphenolic compounds for each cultivar was performed using chromatography data, MS/MS fragmentation patterns, exact mass, and isotopic profiles, aiming to highlight cultivars with valuable phytochemical traits for use in forage improvement, ecological restoration, or potential nutraceutical development.

## 2 Materials and methods

### 2.1 Plant material and sample treatment

All *Lotus* cultivars were produced and/or maintained by the National Institute of Agricultural Research (INIA), located in Tacuarembó, Uruguay. Five of these cultivars belong to the *L. corniculatus*: cultivars San Gabriel, INIA Rigel, and INIA Draco, and experimental lines from rhizomatous *L. corniculatus* types, LE304-C1 Bulk 14, and LE304; three to *L. uliginosus*: cultivars INIA E-Tanin, INIA Gemma, and Grasslands Maku; and the remaining two are hybrids resulting from reciprocal crosses between *L. corniculatus* and *L. uliginosus*: experimental lines G5 Bulk 15, and G1 Bulk 15 (Table 1). Ten independent samples were processed for each of the 10 cultivars. The forage samples were collected from cuttings of forage trials planted in different years (2016–2018) and collected in three seasons (fall, spring and summer). In summary, 100 forage samples were analyzed (Supplementary Table S1).

**TABLE 1:** Plant material origin for each species.

Species	Type	Commercial/experimental name
*Lotus corniculatus*	European	INIA Rigel
*Lotus corniculatus*	European	INIA Draco
*Lotus corniculatus*	European	San Gabriel
*Lotus corniculatus*	Rhizomatous	LE304-C1 Bulk14
*Lotus corniculatus*	Rhizomatous	LE304
*Lotus uliginosus*	Tetraploid	INIA Gemma
*Lotus uliginosus*	Tetraploid	Grasslands Maku
*Lotus uliginosus*	Diploid	INIA E-Tanin
*L. uliginosus* x *L. corniculatus*	Hybrid	G1 Bulk 15
*L. corniculatus* x *L. uliginosus*	Hybrid	G5 Bulk 15

Fresh plant material was dried in an air-forced oven at 40 °C for 48 h and ground. For each sample, 15 g was placed in a glass beaker with 150 mL of acetone-water solution (70:30, v/v) and suspended in an ultrasonic water bath (Branson 3210) for 20 min at room temperature. The extract was obtained from the filtered material and the acetone was removed at a temperature below 35 °C using a rotavapor system (Buchi R-215). The aqueous solution was washed four times with 150 mL dichloromethane to remove chlorophyll and lipids. Finally, the phenolic-rich extract was lyophilized (Labconco free zone 2.5®) and kept refrigerated at 4 °C in air-tight containers until its use.

The extracts (20 mg) were dissolved in 1 mL of water: acetonitrile (50:50 v/v) with 0.1% formic acid, filtered using 0.45 µm syringe membrane filters (FILTER-LAB, Barcelona, Spain), and stored in 2 mL amber chromatographic injection vials at 4 °C until LC-HRMS analysis. A quality control (QC) sample was prepared by mixing 50 µL of each one of the re-constituted phenolic-rich extracts. This QC was employed to assess the reproducibility of the proposed UHPLC-HRMS fingerprinting methodology and to ensure the robustness of the chemometric results.

### 2.2 Reagents and general instrumentation

Acetone and dichloromethane of analytical grade were purchased from Merck (Darmstadt, Germany). For chromatographic separation, the following solvents were used: formic acid (≥95%, Sigma-Aldrich, St Louis, USA) and acetonitrile (99.9%, UHPLC Supergradient, Panreac, Barcelona, Spain). Distilled water was purified with a Milli-Q water purification system (Millipore, Bedford, MS, USA). Chromatography was performed on a Dionex Ultimate 3000 Rapid Separation Liquid Chromatography (RSLC) system (Thermo Scientific, San José, CA, USA), equipped with a vacuum degasser, binary high-pressure pump and autosampler coupled to a linear ion trap mass spectrometer LTQ Orbitrap Velos HRMS from Thermo Scientific (San José, CA, USA) with an ESI interface. Instrument control and data collection were done using Xcalibur software (v3.0.63).

### 2.3 UHPLC-HRMS method

The methodology used was an untargeted metabolomics approach based on ultra-high-performance liquid chromatography (UHPLC) coupled to high-resolution mass spectrometry (HRMS) fingerprinting.

The chromatographic separation was performed in reversed-phase mode with a Kinetex® C18 (100 mm length × 4.6 mm I.D., 2.6 μm partially porous particle size) column from Phenomenex (Torrance, CA, USA). The mobile phase consisted of a gradient of water (solvent A) and acetonitrile (solvent B), both with 0.1% formic acid. The gradient elution program was as follows: from 0 to 3 min, the composition was maintained at 3% B; from 3 to 18 min, a linear gradient was applied to reach 30% B; from 18 to 23 min, the gradient continued to 65% B; from 23 to 25 min, it was further adjusted to 90% B; and from 25 to 26 min, the system returned to the initial conditions at 3% B. The column was conditioned for 7 min under these initial conditions before the next injection. The flow rate was 0.7 mL/min, the column temperature 35 °C, and the injection volume 5 μL. The sample injection order was randomized, with QC solution and solvent blanks injected every 10 samples to prevent any signal drift associated with the sample analysis sequence.

For acquisition, ESI source operated in negative ionization mode. Sheath, sweep and auxiliary gases were nitrogen, with a purity higher than 99,98%, at flow rates of 60, 0 and 10 a.u. (arbitrary units), respectively. The capillary and ESI ionization source temperatures were 350 °C and 25 °C, respectively, and an S-Lens RF level of 50 V was employed. The mass spectrometer operated in data-dependent scan mode, with each cycle including a full MS scan from m/z 100 to 1500, followed by MS/MS scans of the most abundant ions (with full scan signal higher than 1 × 10^5^). HRMS acquisition was performed at 60,000 full width at half-maximum (FWHM, at *m/z* 200) resolution. An automatic gain control (AGC) of 1 ×10^6^, and a maximum injection time (IT) of 200 ms were also employed. A commercially available calibration solution (Thermo Fisher Scientific) was employed for the tuning and calibration of the linear ion-trap (LTQ)-Orbitrap Velos HRMS instrument.

### 2.4 Data analysis

UHPLC-HRMS raw chromatographic data were then processed using the MZmine-2.53 free software to obtain a matrix (sample by variables) of ion signal intensity values, where variables corresponded to each ion feature, characterized by *m/z* and chromatographic retention time.

First, mass detection was performed to generate mass lists for each MS scan, applying a noise level threshold of 2 × 10^4^. These lists were then refined using the FTMS shoulder peak filter to eliminate spurious signals, employing a Gaussian peak shape model and a resolution setting of 70,000. The chromatogram builder module was then applied to link signals detected in consecutive scans, using a *m/z* tolerance of 5 ppm, a retention time range of 5–20 min, and a minimum intensity of 2 × 10^4^. To isolate individual chromatographic peaks, chromatogram deconvolution was performed. Alignment across samples was carried out using the Join Aligner function, with parameters set at 5 ppm for mass tolerance, 500 for weight for *m/z*, 1 min for retention time tolerance, and 10 for weight for retention time. The final aligned feature table was exported in CSV format.

Following this, LC-HRMS fingerprints were filtered to remove spurious features that appeared sporadically in a few samples and did not follow a general pattern; only those detected in, at least, five samples were retained in the data matrix. The resulting matrix was used for the chemometric study and had dimensions (samples + QCs × variables) of 110 × 1929.

Principal component analysis (PCA) and partial least squares-discriminant analysis (PLS-DA) were performed using SOLO 8.6 chemometrics software (Eigenvector Research, Manson, WA, USA). Unsupervised PCA was used to explore the distribution of the analyzed *Lotus* cultivars and to assess the behavior of the injected QCs. Thereafter, PLS-DA was applied for classification purposes, defining three sample sets: (1) interspecies comparison between *L. uliginosus*, *L. corniculatus*, and the two hybrids, forming four groups or classes; (2) intraspecies comparison among the three cultivars of *L. uliginosus* (three groups or classes); and (3) intraspecies comparison among the five cultivars of *L. corniculatus* (five groups or classes).

The X-data matrix used for PCA consisted of the nontargeted LC-HRMS metabolomic fingerprints obtained from all analyzed *Lotus* samples and QCs. For PLS-DA, the same X-data matrix, excluding the QCs, was used, but sample sets were analyzed separately. The Y-data matrix defined the sample classes according to the evaluated case (sets 1–3). The number of latent variables (LVs) used in PLS-DA was determined based on the first relevant minimum of the cross-validation (CV) error using a Venetian blinds approach.

Variable importance in projection (VIP) values obtained from the PLS-DA models for each sample set were used to select the variables with the greatest influence on the distribution of the samples, using a cut-off value of 1.5. Based on these selected variables, metabolite identification was carried out using chromatographic data, HRMS/MS spectra, exact mass, and isotopic pattern data. Then, a heatmap was generated using the *pheatmap* package in R (R Core Team, 2023), based on the previously identified metabolites. Hierarchical clustering was performed using Pearson correlation.

In addition, chemical characterization of the phenolic-rich extracts from the ten studied cultivars was performed. The same strategy was used for this purpose, relying on chromatographic and high-resolution mass spectrometry (HRMS/MS) data.

## 3 Results

### 3.1 LC-HRMS fingerprints

**FIGURE 1:**
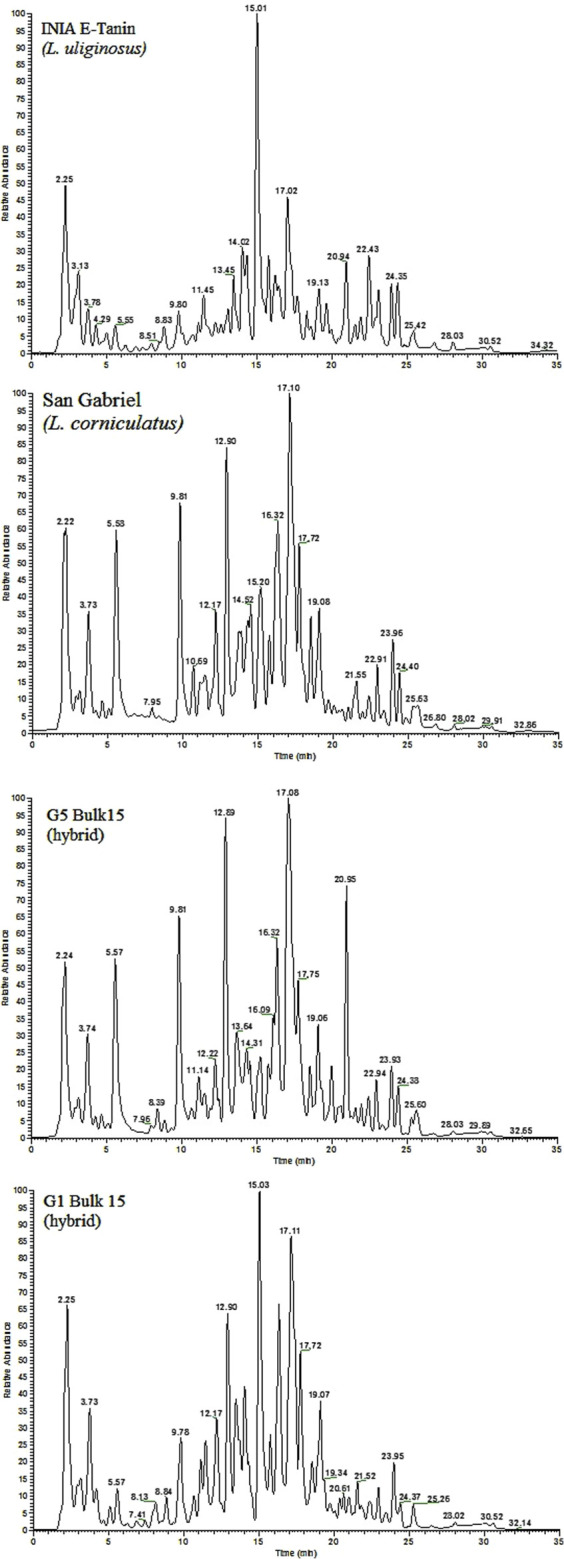
LC-HRMS fingerprints (Total ion chromatograms) for four selected *Lotus* samples.

The whole analytical strategy was focused on obtaining a fingerprint strongly related to phenolic compounds: extracts enriched in phenolic compounds, chromatographic separation based on a C18 column and water and acetonitrile (both acidified with 0.1% of formic acid) as the mobile phase components, and spray ionization (ESI) in negative mode. Figure 1 shows the fingerprints of four selected *Lotus* samples as an example. The total ion chromatograms (TICs) include one from a sample of *L. uliginosus*, another from *L. corniculatus*, and the other two from different *Lotus* hybrids, respectively. Notably, significant differences in both the number of detected peaks and their intensities are observed among the different *Lotus* cultivars. Most of these peaks correspond to phenolic compounds and could be tentatively identified, as will be discussed later. However, in this study, we aimed to apply a fingerprinting approach, using total ion chromatograms as chemical descriptors to facilitate the classification of the samples.

### 3.2 Principal component analysis (PCA) and partial least squares discriminant analysis (PLS-DA)

The matrix obtained from MZmine software includes the non-targeted UHPLC-HRMS metabolomics fingerprints of 100 analyzed *Lotus* samples and the QCs. This data matrix was then submitted to PCA. QCs were well clustered, revealing the good performance of the proposed UHPLC-HRMS methodology as well as the feasibility of the obtained chemometric results. The samples tend to be grouped into 4 groups: *L. uliginosus* species, G1 Bulk 15 (hybrid), *L. corniculatus* species, and G5 Bulk 15 (hybrid).

**FIGURE 2:**
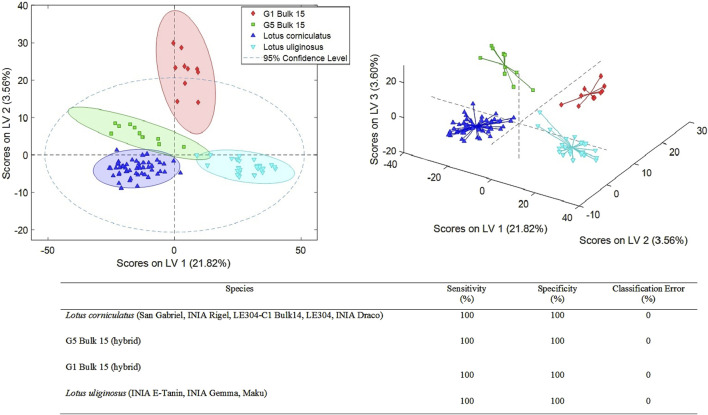
PLS-DA score plots (LV1 vs. LV2 and LV1 vs. LV2 vs. LV3) of a classification model using 6 LVs. Sensitivity, specificity and classification error values obtained when studying the classifications of the analyzed *Lotus* by species.

The UHPLC-HRMS fingerprints were also submitted to PLS-DA to perform a supervised sample classification in each of the three defined cases (sample sets 1–3). The X-data matrix was changed according to the set of samples to be studied and without including QCs. When we analyze sample set 1 which corresponds to the interspecies comparison between *L. uliginosus*, *L. corniculatus*, and the two hybrids, a total of six latent variables (LVs) were required for the optimal PLS-DA model. This model achieved 100% classification accuracy, with sensitivity and specificity values both reaching 100% (Figure 2).

**FIGURE 3:**
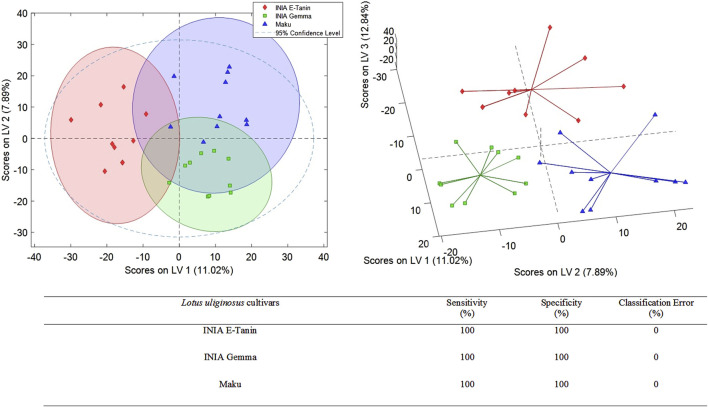
PLS-DA score plots (LV1 vs. LV2 and LV1 vs. LV2 vs. LV3) of a classification model using 4 LVs. Sensitivity, specificity and classification error values obtained when studying the classifications of the analyzed *Lotus uliginosus.*

**FIGURE 4:**
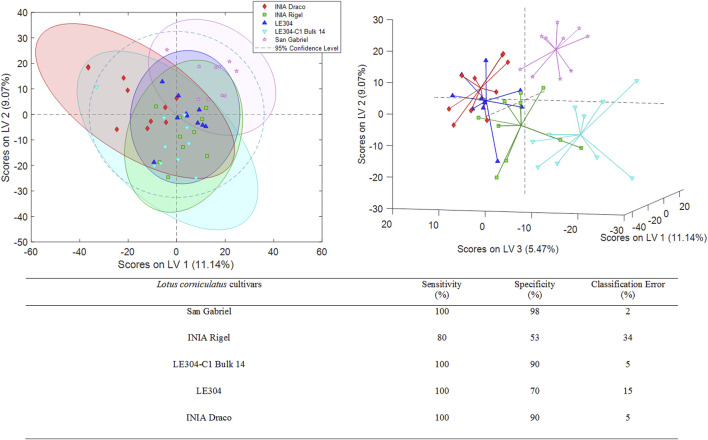
PLS-DA score plots (LV1 vs. LV2 and LV1 vs. LV2 vs. LV3) of a classification model using 6 LVs. Sensitivity, specificity and classification error values obtained when studying the classifications of the analyzed *Lotus corniculatus*.

Additional PLS-DA models were developed to assess whether the 3 *L. uliginosus* cultivars (sample set 2) and the 5 *L. corniculatus* cultivars (sample set 3) could be distinguished from each other, respectively. The best PLS-DA classification score plots obtained for sets 2 and 3 are shown in Figures 3, 4. For sample set 2, the discrimination among the 3 cultivars of *L. uliginosus* is clear. Cross-validated multiclass predictions yielded 100% sensitivity and specificity, with complete (100%) classification accuracy. In contrast, for the classification of *L. corniculatus* cultivars, the proposed approach produced moderately acceptable results, with varying levels of discrimination, as summarized in Figure 4. Among the *L. corniculatus* cultivars, San Gabriel, LE304-C1 Bulk 14, and INIA Draco achieved 100% sensitivity and specificity values equal to or above 90%, with classification errors equal to or below 5.0%. On the other hand, discrimination was less successful for the other two cultivars (INIA Rigel and LE304) with INIA Rigel showing the poorest performance, reaching sensitivity and specificity values of 80% and 53%, respectively, and a classification error of 34%.

### 3.3 Heatmaps analysis

**FIGURE 5:**
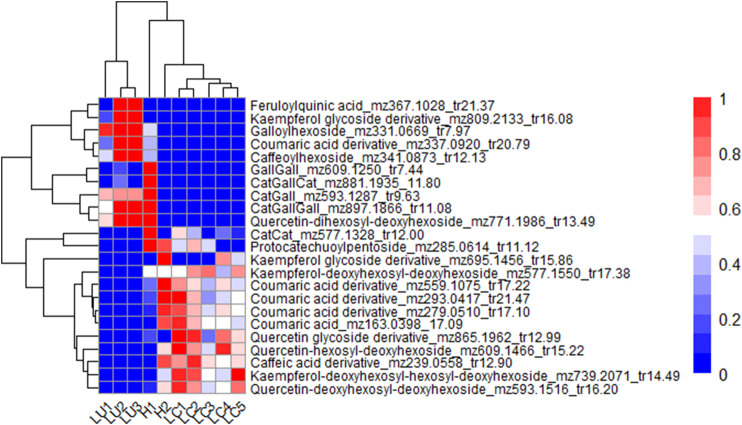
Heatmap showing the most relevant metabolites differentiating *L. uliginosus*, *L. corniculatus*, and the hybrids G5 Bulk 15 and G1 Bulk 15. The x-axis displays the clustering of all *Lotus samples*, while the y-axis shows the clustering of the metabolites. Each *Lotus cultivars* on the x-axis represents the average of 10 independent samples analyzed. LU1: *L. uliginosus* INIA E-Tanin; LU2: *L. uliginosus* INIA Gemma; LU3: *L. uliginosus* Maku; H1: Hybrid G1 Bulk 15; H2: Hybrid G5 Bulk 15; LC1: *L. corniculatus* San Gabriel; LC2: *L. corniculatus* LE304; LC3: *L. corniculatus* INIA Draco; LC4: *L. corniculatus* INIA Rigel; LC5: *L. corniculatus* LE304-C1 Bulk 14.

The main metabolites distinguishing the *Lotus* species in sample set 1 were twenty-three phenolic compounds obtained from PLS-DA VIP analysis, all with VIP values greater than or equal to 1.5. These included phenolic acids, flavonol glycosides, and proanthocyanidins (PAs). Figure 5 presents the hierarchical clustering and heatmap, highlighting differences in the abundance of these phenolic compounds across the *Lotus* samples. The dendrogram on the y-axis reflects the similarity-based clustering of the differential metabolites. Two major metabolite groups can be broadly distinguished. The first group includes ten compounds that are more abundant in the 3 *L. uliginosus* cultivars and the *Lotus* hybrid G1 Bulk 15. These consist of two flavonol glycosides: a kaempferol glycoside derivative (*m/z* 809.2133, *tr* 16.08 min) and a quercetin dihexosyl-deoxyhexoside (*m/z* 771.1986, *tr* 13.49 min); four phenolic acids: caffeoylhexoside (*m/z* 341.0873, *tr* 12.13 min), a coumaric acid derivative (*m/z* 337.0920, *tr* 20.79 min), galloylhexoside (*m/z* 331.0669, *tr* 7.97 min), and feruloylquinic acid (*m/z* 367.1028, *tr* 21.37 min); and four proanthocyanidins. Of these, three are heterogeneous, composed of catechin or epicatechin and gallocatechin or epigallocatechin units: Cat-Gall (*m/z* 593.1287, *tr* 9.63 min), Cat-Gall-Cat (*m/z* 881.1935, *tr* 11.80 min), and Cat-Gall-Gall (*m/z* 897.1866, *tr* 11.08 min). The fourth is a homogeneous prodelphinidin composed exclusively of gallocatechin or epigallocatechin units: Gall-Gall (*m/z* 609.1250, *tr* 7.44 min). The second group also consists of ten metabolites but is characterized by lower abundance in *L. uliginosus* and G1 Bulk 15. This set is composed exclusively of flavonol glycosides and phenolic acids. The flavonol aglycones are quercetin and kaempferol, each conjugated with various monosaccharide residues. The identified glycosides include: a kaempferol glycoside derivative (*m/z* 695.1456, *tr* 15.86 min), a quercetin glycoside derivative (*m/z* 865.1962, *tr* 12.99 min), quercetin hexosyl-deoxyhexoside (*m/z* 609.1466, *tr* 15.22 min), kaempferol deoxyhexosyl-hexosyl-deoxyhexoside (*m/z* 739.2071, *tr* 14.49 min), and quercetin deoxyhexosyl-deoxyhexoside (*m/z* 593.1516, *tr* 16.20 min). The phenolic acids in this group include a caffeic acid derivative (*m/z* 239.0558, *tr* 12.90 min), coumaric acid (*m/z* 163.0398, *tr* 17.09 min), and three additional coumaric acid derivatives: *m/z* 559.1075 (*tr* 17.22 min), *m/z* 293.0417 (*tr* 21.47 min), and *m/z* 279.0510 (*tr* 17.10 min). In addition, three compounds were found to be uniquely less abundant in *L. uliginosus*, while the hybrid G1 Bulk 15 did not share this pattern. These were identified as procyanidin Cat-Cat (*m/z* 577.1328, *tr* 12.00 min), a protocatechuic acid derivative (*m/z* 285.0614, *tr* 11.12 min), and kaempferol deoxyhexosyl-deoxyhexoside (*m/z* 577.1550, *tr* 17.38 min). Finally, the x-axis dendrogram groups the *Lotus* samples based on their metabolite profiles. The heatmap clearly distinguishes two major clusters: one comprising the 3 *L. uliginosus* cultivars and the hybrid G1 Bulk 15, and another including the 5 *L. corniculatus* cultivars and the hybrid G5 Bulk 15. Within these clusters, sub-groupings are observed among the different cultivars of both species.

**FIGURE 6:**
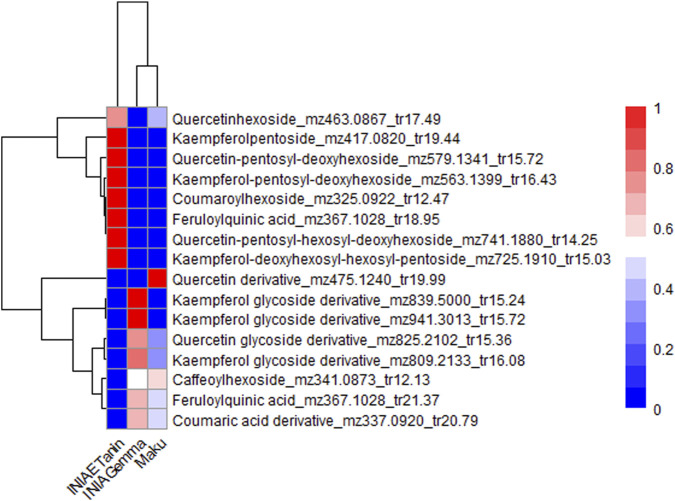
Heatmap showing the most relevant metabolites differentiating *Lotus uliginosus* cultivars. The x-axis displays the clustering of all *Lotus* cultivars, while the y-axis shows the clustering of the metabolites. Each cultivar on the x-axis represents the average of 10 independent samples analyzed.

**FIGURE 7:**
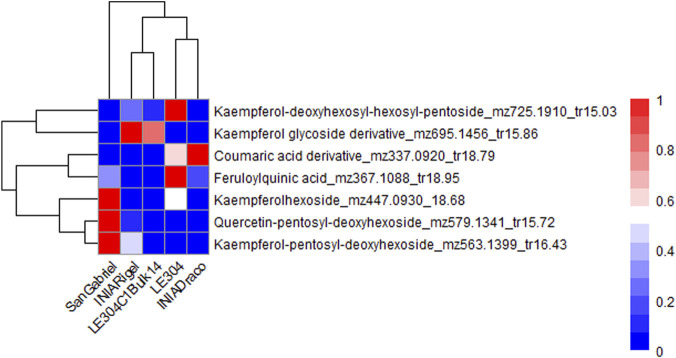
Heatmap showing the most relevant metabolites differentiating *Lotus corniculatus* cultivars. The x-axis displays the clustering of all *Lotus* cultivars, while the y-axis shows the clustering of the metabolites. Each cultivar on the x-axis represents the average of 10 independent samples analyzed.

Variable Importance in Projection (VIP) values were also obtained from the PLS-DA models for sample sets 2 and 3. In the comparison between *L. uliginosus* cultivars (sample set 2) and *L. corniculatus* cultivars (sample set 3), sixteen and seven metabolites with VIP values greater than 1.5 were identified, respectively. These metabolites were also phenolic compounds, including flavonol glycosides and phenolic acids. In both cases, proanthocyanidins (PAs) were not significant contributors to the differentiation between the cultivars of each of the species. Figures 6, 7 show the hierarchical clustering and corresponding heatmaps, highlighting differences in the abundance of these phenolic compounds across *L. uliginosus* and *L. corniculatus*, respectively.

INIA Gemma and Grasslands Maku, both *L. uliginosus* cultivars, are clustered together in the heatmaps of sample sets 1 and 2, despite being based on different sets of metabolites, except for three compounds that are common to both. Similarly, INIA Rigel and LE304 C1 Bulk 14, both *L. corniculatus* cultivars, are grouped together in the heatmaps of sets 1 and 3.

### 3.4 Chemical characterization of phenolic rich-extracts of 10 *Lotus* cultivars from Uruguay

An additional objective of this study was to characterize a broader range of phenolic compounds in these cultivars, thereby contributing to a more comprehensive understanding of the phenolic composition in *Lotus* species since they define various properties.

The compounds contained in cultivar extracts were adequately separated by chromatography on a C-18 reverse-phase UHPLC column using a mobile phase gradient of acetonitrile in water at acidic conditions. Figure 1 provides an overview of the total ion chromatograms obtained from selected extracts, highlighting their complex chemical profiles. The mass spectrometer was operated to monitor, select and fragment the most abundant ions within the *m/z* 100–1500 range. Metabolite identification was based on chromatography data, HRMS/MS spectra, exact mass measurements (máximum error of mass 5 ppm), and isotopic pattern data. Additionally, the NIST 20 Tandem Library (hr_msms_nist and nist_msms) was consulted using the MS/MS Identity Search function of the NIST MS Search Program (v.2.4).

Under this approach, 105 compounds were tentatively identified, including phenolic acids, flavonols, flavan-3-ols, proanthocyanidins, and other organic acids. The assigned compounds, along with their chromatographic and mass spectral data, are listed in Supplementary Table S2. When a phenolic compound is labelled in the table as not detected [marked in yellow (−)], it means that it was not detected in all ten analyzed samples within the same *Lotus* cultivar. In contrast, when a compound is labelled as detected [marked in green (+)], it was, on average, present in most of the ten analyzed samples in that cultivar, although it was not present in some individual samples.

#### 3.4.1 Identification of organic acids and their derivatives

A total of thirty-seven organic acids were tentatively identified, the majority of which were phenolic acids (thirty-two), including hydroxybenzoic and hydroxycinnamic acids. The hydroxybenzoic acids identified included protocatechuic acid (in free, glycosylated, and derivative forms), gallic acid (in its free form and as a hexoside), hydroxybenzoylhexoside, vanilloylhexoside, and syringoylhexoside. All these compounds were detected across the ten *Lotus* cultivars analyzed, except for syringoylhexoside, which was not found in any of the independent samples of the INIA Rigel cultivar. A total of twenty-three hydroxycinnamic acids were identified, some of which appeared in two or three isomeric forms. Others were glycosylated, and some were derivatives for which a specific molecular structure could not be proposed. For example, compounds 10 and 11 in Supplementary Table S2 were identified as isomers of coumaric acid. In addition, the hexoside of coumaric acid and seven coumaric acid derivatives were also identified. All coumaric acid derivatives exhibited, in their HRMS/MS spectra, the characteristic ions of coumaric acid and its decarboxylated product, at *m/z* 163.0398 and 119.0502, respectively. Chlorogenic acid isomers were identified at retention times of 10.76, 13.30, and 14.03 min, each displaying a [M–H]^-^ ion at *m/z* 353.0874 with a mass error of 0.397 ppm. These compounds are formed by the esterification of caffeic acid with quinic acid and differ based on the position of the ester linkage: 3-O-caffeoylquinic acid (3-CQA), 4-O-caffeoylquinic acid (also known as cryptochlorogenic acid or 4-CQA), and 5-O-caffeoylquinic acid (neochlorogenic acid or 5-CQA) (Aguiar et al., 2016). Other organic acids identified included quinic acid, malic acid, isomers of isopropyl malic acid, and a derivative of isopropyl malic acid. The identification of malic acid and its derivatives is consistent with previously reported data for compounds in the *Lotus* genus (Sanchez et al., 2008).

#### 3.4.2 Identification of flavan-3-oles

Four flavan-3-ol compounds were identified: catechin, gallocatechin, and their corresponding isomers, epicatechin and epigallocatechin. In some *Lotus* cultivars, catechin or epicatechin was also detected in glycosylated form, specifically as a hexoside, with a retention time of 10.38 min and a [M–H]^-^ ion at *m/z* 451.1238.

#### 3.4.3 Identification of *flavonols*

Several flavonol glycosides were identified, with quercetin, kaempferol, myricetin, and isorhamnetin as their aglycones. The most abundant aglycones were kaempferol and quercetin. Thirteen glycosides of kaempferol were detected, containing up to three monosaccharide residues, while fourteen glycosides of quercetin were identified, with up to four monosaccharide residues. Depending on the glycoside, characteristic neutral losses of the sugar moieties were observed in the HRMS/MS spectra: pentose (Δ*m/z* = 132), deoxyhexose (Δ*m/z* = 146), hexose (Δ*m/z* = 162), and glucuronide (Δm/z = 176). In some compounds (54, 62, 64, and 65), the monosaccharide residues were acetylated, as evidenced by the neutral loss corresponding to the acetate group (Δ*m/z* = 42) in the HRMS/MS spectra. Additionally, nine kaempferol glycoside derivatives and six quercetin glycoside derivatives were identified.

#### 3.4.4 Identification *of* proanthocyanidins

Homogeneous proanthocyanidins or condensed tannins formed exclusively by (epi) catechin monomers (procyanidins) and by (epi) gallocatechin monomers (prodelphinidins) were identified, as well as heterogeneous formed by (epi) catechin and (epi) gallocatechin units. The chromatographic separation was highly effective, allowing the resolution of several isomeric compounds sharing the same molecular ion (*m/z* 577.1341; 593.1287; 609.1250; 897.1866, and 913.1818).

## 4 Discussion

*Lotus corniculatus* and *L. uliginosus* are species with not only very different agronomic features in terms of habit of growth, environmental niche, botanical traits, and productivity, but also in terms of condensed tannin content (Ayala and Carámbula, 2009). The chemical profiles arising from this technique show very distinguishable differences in the compounds between these species (Figure 1). *Lotus corniculatus* and *L. uliginosus* have high agronomic value in Uruguay. Persistence is generally one of the breeding goals in *L. corniculatus* (Rebuffo and Altier, 1997; Altier et al., 2000), while seed production is the main problem in *L. uliginosus* (Sanders and Rowarth, 1995). Inter-specific hybridization opens an opportunity to combine positive attributes from both species, with the objective of widening the genetic basis for breeding and achieving a superior agronomic cultivar. Reciprocal crosses between *L. uliginosus* “INIA Gemma” and *L. corniculatus* “INIA Draco” were performed to obtain hybrids, through embryo rescue (Castillo et al., 2012). From the early beginning, the hybrids showed a strong maternal effect in terms of the phenotype of the plants. After obtaining the first hybrid individuals, an extra cycle of selection was performed selecting by agronomic traits but keeping the two groups depending on the origin of the hybrids, where the hybrid population G1Bulk 15 represents hybrid plants originating from the cross *L. uliginosus* (as the mother) x *L. corniculatus* (as the pollen donor), and hybrid population G5 Bulk 15 is the result of the reciprocal cross (*L. corniculatus* x *L. uliginosus*). Analyzing chemical results from the hybrids (Figure 1), it looks clear that G1 hybrid not only looks like *L. uliginosus* in its phenotype but also in its chemical profile, while a similar pattern is observed for hybrid G5 and *L. corniculatus*.

This methodology was successful to discriminate the main two species, *L. corniculatus* and *L. uliginosus*, and the two hybrid populations. The five cultivars of *L. corniculatus*, were grouped together based on their chemical profile with 0% error rate. Similar results were observed for the 3 *L. uliginosus* cultivars and the two hybrids (Figure 2).

In the case of *L. uliginosus*, the 10 samples from each cultivar were classified as the cultivars that belonged with a 0% error rate, showing the high precision of this approach (Figure 3). *Lotus corniculatus* classification (Figure 4) displays higher error rates. This may be related to the similar genetic origin of some of the cultivars. For instance, INIA Rigel is a reselection of INIA Draco, while LE304-C1 Bulk 14 derives from LE304 and, therefore, genetically related.

Condensed tannins, a class of secondary metabolites characteristic of the *Lotus* genus, played a critical role in interspecies discrimination. These compounds are known to have beneficial effects on animal production, as they enhance intestinal protein absorption, improve animal health, and may contribute to the reduction of methane emissions (Verma et al., 2022; Brutti et al., 2023). Their prominent role in distinguishing species may reflect underlying ecological or adaptive divergence. However, their influence on intraspecies separation was null, suggesting that other components of the phenolic profile are responsible for cultivar-level differentiation. Therefore, while condensed tannins are informative at the species level, comprehensive metabolomic analyses are essential to fully understand the metabolic landscape and potential functional implications of the different *Lotus* cultivars.

Of the 105 phenolic compounds tentatively identified through chromatographic and high-resolution MS/MS data, several were annotated as putative derivatives of known compounds, for which no definitive molecular formula could be assigned. This was particularly the case for compounds related to coumaric acid, which appeared to contribute significantly to interspecies separation. Given their apparent relevance, structural elucidation of these compounds using complementary techniques, such as nuclear magnetic resonance (NMR) spectroscopy, would be highly valuable.

This study provides an in-depth characterization of phenolic compounds in *Lotus* cultivars used in Uruguay, in contrast to many previous works that rely on non-specific determinations of total phenolics, total tannins, or total flavonoids (Montossi, 1996; Reyno et al., 2022). By applying high-resolution LC-MS/MS and integrating metabolomic data with chemometric tools, we highlight the complexity and diversity of phenolic profiles in this genus. These findings open new avenues for investigating the biological functions and potential applications of specific metabolites in forage quality, plant resilience, and beyond.

## 5 Conclusion

In the present study, a non-targeted LC-HRMS metabolomic fingerprinting approach proved the effectiveness for characterizing and classifying ten *Lotus* cultivars and experimental lines, developed in Uruguay, through the application of multivariate chemometric methods. Overall, the PLS-DA models showed satisfactory classification performance across the different *Lotus* sample sets. For each model, the VIP scores enabled the identification of the most discriminant phenolic compounds.

The proposed methodology demonstrated high potential for the tentative identification of polyphenolic compounds, thanks to the accurate mass detection provided by HRMS and the comprehensive chemical information captured by the fingerprinting approach.

These findings underscore the applicability of metabolomic fingerprinting as a powerful and accessible tool in plant breeding programs, particularly for selecting *Lotus* cultivars with favorable phytochemical profiles related to forage quality, nutritional value, or environmental adaptability.

## Data Availability

The original contributions presented in the study are publicly available. This data can be found here: https://doi.org/10.5281/zenodo.16905263.
