# Supplementary material for: LC-HRMS fingerprinting and chemometrics for the characterization and classification of Lotus cultivars from Uruguay: a study on phenolic composition
Source: Front Mol Biosci. 2025 Aug 29;12:1646758. doi: 10.3389/fmolb.2025.1646758 (PMC12425797; doi:10.3389/fmolb.2025.1646758)
Supplement: Supplementary file 1 [file Supplementaryfile1.docx]

Supplementary Material

**Supplementary Figure S1.** Some phenolic compounds typical of plants.


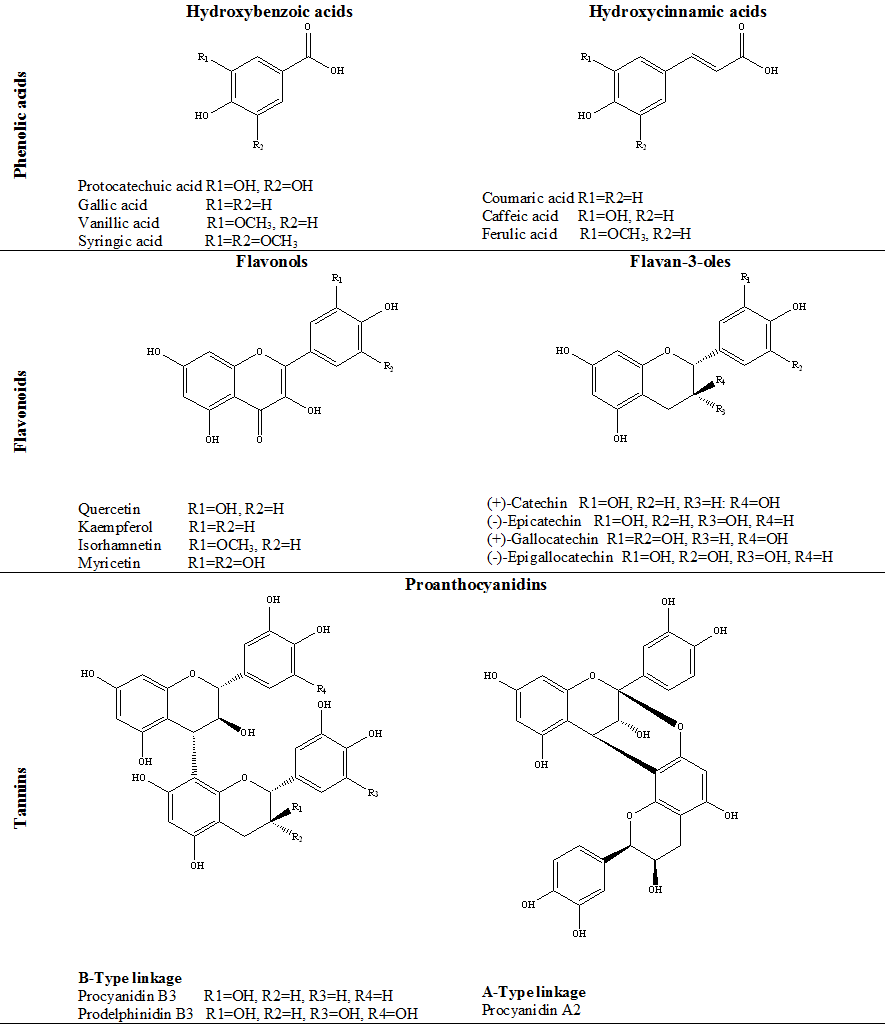


**Supplementary Table S1.** Independent samples for each of the 10 *Lotus* cultivars.

| **Sample code** | **Species** | **Cultivar/Exp. Line** | **Trial**  **code** | **Cutting**  **date** |
| --- | --- | --- | --- | --- |
| 1-1 | *L. corniculatus* | San Gabriel | 1603 | Spring-2018 |
| 1-2 | *L. corniculatus* | San Gabriel | 1603 | Spring-2018 |
| 1-3 | *L. corniculatus* | San Gabriel | 1703 | Spring-2018 |
| 1-4 | *L. corniculatus* | San Gabriel | 1703 | Spring-2018 |
| 1-5 | *L. corniculatus* | San Gabriel | 1703 | Summer-2019 |
| 1-6 | *L. corniculatus* | San Gabriel | 1803 | Spring-2018 |
| 1-7 | *L. corniculatus* | San Gabriel | 1803 | Spring-2018 |
| 1-8 | *L. corniculatus* | San Gabriel | 1803 | Summer-2019 |
| 1-9 | *L. corniculatus* | San Gabriel | 1703 | Spring-2018 |
| 1-10 | *L. corniculatus* | San Gabriel | 1703 | Spring-2018 |
| 2-1 | *L. corniculatus x L. uliginosus* | G5 Bulk15 | 1603 | Spring-2018 |
| 2-2 | *L. corniculatus x L. uliginosus* | G5 Bulk15 | 1603 | Spring-2018 |
| 2-3 | *L. corniculatus x L. uliginosus* | G5 Bulk15 | 1603 | Summer-2019 |
| 2-4 | *L. corniculatus x L. uliginosus* | G5 Bulk15 | 1703 | Spring-2018 |
| 2-5 | *L. corniculatus x L. uliginosus* | G5 Bulk15 | 1703 | Summer-2019 |
| 2-6 | *L. corniculatus x L. uliginosus* | G5 Bulk15 | 1703 | Fall-2019 |
| 2-7 | *L. corniculatus x L. uliginosus* | G5 Bulk15 | 1703 | Spring-2018 |
| 2-8 | *L. corniculatus x L. uliginosus* | G5 Bulk15 | 1703 | Spring-2018 |
| 2-9 | *L. corniculatus x L. uliginosus* | G5 Bulk15 | 1703 | Spring-2018 |
| 2-10 | *L. corniculatus x L. uliginosus* | G5 Bulk15 | 1703 | Spring-2018 |
| 3-1 | *L. uliginosus* | INIA E-Tanin | 1603 | Spring-2018 |
| 3-2 | *L. uliginosus* | INIA E-Tanin | 1703 | Spring-2018 |
| 3-3 | *L. uliginosus* | INIA E-Tanin | 1703 | Summer-2019 |
| 3-4 | *L. uliginosus* | INIA E-Tanin | 1703 | Fall-2019 |
| 3-5 | *L. uliginosus* | INIA E-Tanin | 1803 | Spring-2018 |
| 3-6 | *L. uliginosus* | INIA E-Tanin | 1803 | Spring-2018 |
| 3-7 | *L. uliginosus* | INIA E-Tanin | 1803 | Summer-2019 |
| 3-8 | *L. uliginosus* | INIA E-Tanin | 1703 | Spring-2018 |
| 3-9 | *L. uliginosus* | INIA E-Tanin | 1703 | Spring-2018 |
| 3-10 | *L. uliginosus* | INIA E-Tanin | 1703 | Spring-2018 |
| 4-1 | *L. corniculatus* | INIA Rigel | 1603 | Spring-2018 |
| 4-2 | *L. corniculatus* | INIA Rigel | 1603 | Spring-2018 |
| 4-3 | *L. corniculatus* | INIA Rigel | 1703 | Spring-2018 |
| 4-4 | *L. corniculatus* | INIA Rigel | 1703 | Spring-2018 |
| 4-5 | *L. corniculatus* | INIA Rigel | 1803 | Spring-2018 |
| 4-6 | *L. corniculatus* | INIA Rigel | 1803 | Summer-2019 |
| 4-7 | *L. corniculatus* | INIA Rigel | 1703 | Spring-2018 |
| 4-8 | *L. corniculatus* | INIA Rigel | 1703 | Spring-2018 |
| 4-9 | *L. corniculatus* | INIA Rigel | 1803 | Fall-2019 |
| 4-10 | *L. corniculatus* | INIA Rigel | 1703 | Spring-2018 |
| 5-1 | *L. corniculatus* | LE304-C1 Bulk 14 | 1603 | Spring-2018 |
| 5-2 | *L. corniculatus* | LE304-C1 Bulk 14 | 1603 | Spring-2018 |
| 5-3 | *L. corniculatus* | LE304-C1 Bulk 14 | 1603 | Summer-2019 |
| 5-4 | *L. corniculatus* | LE304-C1 Bulk 14 | 1703 | Summer-2019 |
| 5-5 | *L. corniculatus* | LE304-C1 Bulk 14 | 1803 | Spring-2018 |
| 5-6 | *L. corniculatus* | LE304-C1 Bulk 14 | 1803 | Spring-2018 |
| 5-7 | *L. corniculatus* | LE304-C1 Bulk 14 | 1803 | Summer-2019 |
| 5-8 | *L. corniculatus* | LE304-C1 Bulk 14 | 1803 | Fall-2019 |
| 5-9 | *L. corniculatus* | LE304-C1 Bulk 14 | 1703 | Spring-2018 |
| 5-10 | *L. corniculatus* | LE304-C1 Bulk 14 | 1703 | Spring-2018 |
| 6-1 | *L. uliginosus x L. corniculatus* | G1 Bulk 15 | 1603 | Spring-2018 |
| 6-2 | *L. uliginosus x L. corniculatus* | G1 Bulk 15 | 1603 | Spring-2018 |
| 6-3 | *L. uliginosus x L. corniculatus* | G1 Bulk 15 | 1703 | Spring-2018 |
| 6-4 | *L. uliginosus x L. corniculatus* | G1 Bulk 15 | 1703 | Summer-2019 |
| 6-5 | *L. uliginosus x L. corniculatus* | G1 Bulk 15 | 1703 | Fall-2019 |
| 6-6 | *L. uliginosus x L. corniculatus* | G1 Bulk 15 | 1803 | Spring-2018 |
| 6-7 | *L. uliginosus x L. corniculatus* | G1 Bulk 15 | 1803 | Fall-2019 |
| 6-8 | *L. uliginosus x L. corniculatus* | G1 Bulk 15 | 1703 | Spring-2018 |
| 6-9 | *L. uliginosus x L. corniculatus* | G1 Bulk 15 | 1703 | Spring-2018 |
| 6-10 | *L. uliginosus x L. corniculatus* | G1 Bulk 15 | 1703 | Spring-2018 |
| 7-1 | *L. uliginosus* | INIA Gemma | 1603 | Spring-2018 |
| 7-2 | *L. uliginosus* | INIA Gemma | 1603 | Spring-2018 |
| 7-3 | *L. uliginosus* | INIA Gemma | 1603 | Summer-2019 |
| 7-4 | *L. uliginosus* | INIA Gemma | 1703 | Summer-2019 |
| 7-5 | *L. uliginosus* | INIA Gemma | 1703 | Spring-2018 |
| 7-6 | *L. uliginosus* | INIA Gemma | 1803 | Summer-2019 |
| 7-7 | *L. uliginosus* | INIA Gemma | 1703 | Spring-2018 |
| 7-8 | *L. uliginosus* | INIA Gemma | 1703 | Spring-2018 |
| 7-9 | *L. uliginosus* | INIA Gemma | 1703 | Spring-2018 |
| 7-10 | *L. uliginosus* | INIA Gemma | 1703 | Spring-2018 |
| 8-1 | *L. corniculatus* | LE304 | 1603 | Spring-2018 |
| 8-2 | *L. corniculatus* | LE304 | 1603 | Spring-2018 |
| 8-3 | *L. corniculatus* | LE304 | 1703 | Spring-2018 |
| 8-4 | *L. corniculatus* | LE304 | 1703 | Spring-2018 |
| 8-5 | *L. corniculatus* | LE304 | 1703 | Spring-2018 |
| 8-6 | *L. corniculatus* | LE304 | 1703 | Summer-2019 |
| 8-7 | *L. corniculatus* | LE304 | 1803 | Spring-2018 |
| 8-8 | *L. corniculatus* | LE304 | 1803 | Summer-2019 |
| 8-9 | *L. corniculatus* | LE304 | 1803 | Fall-2019 |
| 8-10 | *L. corniculatus* | LE304 | 1803 | Summer-2019 |
| 9-1 | *L. uliginosus* | Grasslands Maku | 1603 | Summer-2019 |
| 9-2 | *L. uliginosus* | Grasslands Maku | 1703 | Spring-2018 |
| 9-3 | *L. uliginosus* | Grasslands Maku | 1603 | Spring-2018 |
| 9-4 | *L. uliginosus* | Grasslands Maku | 1603 | Spring-2018 |
| 9-5 | *L. uliginosus* | Grasslands Maku | 1703 | Spring-2018 |
| 9-6 | *L. uliginosus* | Grasslands Maku | 1703 | Summer-2019 |
| 9-7 | *L. uliginosus* | Grasslands Maku | 1803 | Summer-2019 |
| 9-8 | *L. uliginosus* | Grasslands Maku | 1803 | Fall-2019 |
| 9-9 | *L. uliginosus* | Grasslands Maku | 1803 | Summer-2019 |
| 9-10 | *L. uliginosus* | Grasslands Maku | 1803 | Summer-2019 |
| 10-1 | *L. corniculatus* | INIA Draco | 1703 | Fall-2019 |
| 10-2 | *L. corniculatus* | INIA Draco | 1603 | Spring-2019 |
| 10-3 | *L. corniculatus* | INIA Draco | 1703 | Summer-2019 |
| 10-4 | *L. corniculatus* | INIA Draco | 1703 | Spring-2018 |
| 10-5 | *L. corniculatus* | INIA Draco | 1703 | Spring-2018 |
| 10-6 | *L. corniculatus* | INIA Draco | 1603 | Summer-2019 |
| 10-7 | *L. corniculatus* | INIA Draco | 1703 | Spring-2018 |
| 10-8 | *L. corniculatus* | INIA Draco | 1603 | Spring-2018 |
| 10-9 | *L. corniculatus* | INIA Draco | 1603 | Spring-2019 |
| 10-10 | *L. corniculatus* | INIA Draco | 1603 | Spring-2019 |

**Supplementary Table S2.** Chromatographic and mass spectral data from phenolic-rich extracts obtained from ten cultivars of *Lotus* by LC-HRMS/MS analysis.

| **Phenolic acids: hydroxybenzoic acids(1-9) and hydroxycinnamic acids (10-33)** | | | | | | | | | | | | | | | | | |
| --- | --- | --- | --- | --- | --- | --- | --- | --- | --- | --- | --- | --- | --- | --- | --- | --- | --- |
| **N°** | **tr**  **(min)** | **Exp.**  ***(m/z)*** | **Adduct** | **Formula Molecular**  **(Adduct)** | **Mass error (ppm)** | **Main fragments**  ***(m/z)*** | **Identification** | **INIA E-Tanin**  **(*L. uliginosus)*** | **INIA Gemma**  **(*L. uliginosus)*** | **Grasslands Maku**  **(*L. uliginosus)*** | **San Gabriel**  **(*L. corniculatus)*** | **INIA Rigel**  **(*L. corniculatus)*** | **LE 304-C1 Bulk 14**  **(*L. corniculatus)*** | **LE 304**  **(*L. corniculatus)*** | **INIA Draco**  **(*L. corniculatus)*** | **G5 Bulk 15**  **(hybrid*)*** | **G1 Bulk 15**  **(hybrid)** |
| **1** | **9.97** | **153.0189** | **[M-H]-** | **C_7_H_6_O_4_** | **0,752** | **109** | **Protocatechuic acid** | **+** | **+** | **+** | **+** | **+** | **+** | **+** | **+** | **+** | **+** |
| **2** | **6.30** | **169.0136** | **[M-H]-** | **C_9_H_7_O_3_** | **-0.591** | **125** | **Gallic acid** | **+** | **+** | **+** | **+** | **+** | **+** | **+** | **+** | **+** | **+** |
| **3** | **11.12** | **285.0614** | **[M-H]-** | **C_12_H_13_O_8_** | **1.245** | **153, 109** | **Protocatechuoyl pentoside** | **+** | **+** | **+** | **+** | **+** | **+** | **+** | **+** | **+** | **+** |
| **4** | **11.60** | **299.0761** | **[M-H]-** | **C_13_H_15_O_8_** | **-1.989** | **137, 93** | **hydroxybenzoyl-hexoside** | **+** | **+** | **+** | **+** | **+** | **+** | **+** | **+** | **+** | **+** |
| **5** | **8.20** | **315.0717** | **[M-H]-** | **C_13_H_15_O_9_** | **0.286** | **153, 109** | **Protocatechuoyl hexoside** | **+** | **+** | **+** | **+** | **+** | **+** | **+** | **+** | **+** | **+** |
| **6** | **9.70** | **329.0868** | **[M-H]-** | **C_14_H_17_O_9_** | **-1.398** | **167, 152** | **vanilloylhexoside** | **+** | **+** | **+** | **+** | **+** | **+** | **+** | **+** | **+** | **+** |
| **7** | **7.97** | **331.0669** | **[M-H]-** | **C_13_H_15_O_10_** | **1.133** | **313, 271, 241, 169, 125** | **Galloylhexoside** | **+** | **+** | **+** | **+** | **+** | **+** | **+** | **+** | **+** | **+** |
| **8** | **8.56** | **343.0190** | **-** | **-** | **-** | **299, 153, 125, 109** | **Protocatechuic acid derivative** | **+** | **+** | **+** | **+** | **+** | **+** | **+** | **+** | **+** | **+** |
| **9** | **10.55** | **359.0977** | **[M-H]-** | **C_15_H_19_O_10_** | **-0.348** | **197, 182** | **Syringoylhexoside** | **+** | **+** | **+** | **+** | **-** | **+** | **+** | **+** | **+** | **+** |
| **10** | **11.31** | **163.0398** | **[M-H]-** | **C_9_H_7_O_3_** | **1.717** | **119, 93** | **Coumaric acid**  **isomer I** | **+** | **+** | **+** | **+** | **+** | **+** | **+** | **+** | **+** | **+** |
| **11** | **17.09** | **163.0398** | **[M-H]-** | **C_9_H_7_O_3_** | **1.717** | **119, 93** | **Coumaric acid**  **isomer II** | **+** | **+** | **+** | **+** | **+** | **+** | **+** | **+** | **+** | **+** |
| **12** | **14.08** | **179.0346** | **[M-H]-** | **C_9_H_7_O_4_** | **0.922** | **135** | **Caffeic acid** | **+** | **+** | **+** | **+** | **-** | **+** | **+** | **-** | **+** | **-** |
| **13** | **16.67** | **193.0500** | **[M-H]-** | **C_10_H_9_O_4_** | **-0.440** | **178, 149, 134, 107** | **Ferulic acid** | **+** | **+** | **+** | **+** | **+** | **+** | **+** | **+** | **+** | **+** |
| **14** | **12.90** | **239.0558** | **-** | **-** | **-** | **221, 195, 179, 177, 149, 135, 133, 107** | **Caffeic acid derivative** | **+** | **+** | **+** | **+** | **+** | **+** | **+** | **+** | **+** | **+** |
| **15** | **17.10** | **279.0510** | **-** | **-** | **-** | **163, 133, 119** | **Coumaric acid derivative** | **+** | **+** | **+** | **+** | **+** | **+** | **+** | **+** | **+** | **+** |
| **16** | **21.47** | **293.0417** | **-** | **-** | **-** | **261, 163, 145, 129, 119** | **Coumaric acid derivative** | **-** | **-** | **-** | **+** | **+** | **+** | **+** | **+** | **+** | **+** |
| **17** | **15.10** | **295.0454** | **[M-H]-** | **C_13_H_11_O_8_** | **0.017** | **179. 135, 133, 115** | **Caffeoylmalic acid** | **-** | **-** | **-** | **+** | **+** | **+** | **+** | **+** | **+** | **+** |
| **18** | **11.10** | **311.0404** | **[M-H]-** | **C_13_H_11_O_9_** | **0.289** | **179, 149** | **Caftaric acid** | **+** | **+** | **+** | **+** | **+** | **+** | **+** | **-** | **-** | **-** |
| **19** | **12.47** | **325.0922** | **[M-H]-** | **C_15_H_17_O_8_** | **-0.446** | **163, 119** | **Coumaroylhexoside** | **+** | **+** | **+** | **+** | **+** | **+** | **+** | **+** | **+** | **+** |
| **20** | **16.82** | **325.0922** | **[M-H]-** | **C_15_H_17_O_8_** | **-0.446** | **193, 178, 149, 134, 107** | **Feruloylpentoside** | **+** | **-** | **-** | **+** | **-** | **-** | **+** | **-** | **-** | **-** |
| **21** | **17.40** | **327.0866** | **-** | **-** | **-** | **309, 283, 265, 237, 221, 211, 163, 119** | **Coumaric acid derivative** | **+** | **-** | **-** | **+** | **+** | **+** | **+** | **+** | **+** | **+** |
| **22** | **18.79** | **337.0920** | **-** | **-** | **-** | **277, 223, 191, 163, 119** | **Coumaric acid derivative isomer I** | **+** | **-** | **-** | **-** | **-** | **-** | **+** | **+** | **+** | **+** |
| **23** | **20.79** | **337.0920** | **-** | **-** | **-** | **277, 223, 191, 163, 119** | **Coumaric acid derivative isomer II** | **+** | **+** | **+** | **-** | **-** | **-** | **-** | **-** | **-** | **+** |
| **24** | **12.13** | **341.0873** | **[M-H]-** | **C_15_H_17_O_9_** | **0.117** | **179, 135** | **Caffeoylhexoside** | **+** | **+** | **+** | **-** | **-** | **-** | **-** | **-** | **-** | **-** |
| **25** | **10.76** | **353.0874** | **[M-H]-** | **C_16_H_17_O_9_** | **0,397** | **335, 191, 179, 135** | **Chlorogenic acid isomer I** | **+** | **+** | **+** | **+** | **+** | **+** | **+** | **+** | **+** | **+** |
| **26** | **13.30** | **353.0874** | **[M-H]-** | **C_16_H_17_O_9_** | **0,397** | **335, 191, 179, 135** | **Chlorogenic acid isomer II** | **+** | **+** | **+** | **+** | **+** | **+** | **+** | **+** | **+** | **+** |
| **27** | **14.03** | **353.0874** | **[M-H]-** | **C_16_H_17_O_9_** | **0,397** | **335, 191, 179, 135** | **Chlorogenic acid isomer III** | **+** | **+** | **+** | **-** | **+** | **-** | **-** | **+** | **+** | **-** |
| **28** | **18.95** | **367.1028** | **[M-H]-** | **C_17_H_19_O_9_** | **-0.300** | **307, 193, 191, 178, 134** | **Feruloylquinic acid isomer I** | **+** | **+** | **+** | **-** | **-** | **-** | **-** | **-** | **-** | **-** |
| **29** | **21.37** | **367.1028** | **[M-H]-** | **C_17_H_19_O_9_** | **-0.300** | **307, 193, 191, 178, 134** | **Feruloylquinic acid isomer II** | **+** | **+** | **+** | **-** | **-** | **-** | **-** | **-** | **-** | **-** |
| **30** | **14.70** | **413.1078** | **-** | **-** | **-** | **267, 249, 163, 119** | **Coumaric acid derivative** | **+** | **+** | **+** | **+** | **+** | **+** | **+** | **+** | **+** | **+** |
| **31** | **12.00** | **515.1185** | **[M-H]-** | **C_25_H_23_O_12_** | **-0,883** | **353, 257** | **Quinic acid di-O-caffeate** | **+** | **+** | **+** | **+** | **+** | **+** | **+** | **+** | **+** | **+** |
| **32** | **17.22** | **559.1075** | **-** | **-** | **-** | **443, 327, 309, 283, 239, 191, 163** | **Coumaric acid derivative** | **+** | **+** | **+** | **+** | **+** | **+** | **+** | **+** | **+** | **+** |
| **Other organic acids** | | | | | | | | | | | | | | | | | |
| **N°** | **tr**  **(min)** | **Exp.**  ***(m/z)*** | **Adduct** | **Formula Molecular**  **(Adduct)** | **Mass error (ppm)** | **Main fragments**  ***(m/z)*** | **Identification** | **INIA E-Tanin**  **(*L. uliginosus)*** | **INIA Gemma**  **(*L. uliginosus)*** | **Grasslands Maku**  **(*L. uliginosus)*** | **San Gabriel**  **(*L.corniculatus)*** | **INIA Rigel**  **(*L.corniculatus)*** | **LE 304-C1 Bulk 14**  **(*L.corniculatus)*** | **LE 304**  **(*L.corniculatus)*** | **INIA Draco**  **(*L. corniculatus)*** | **G5 Bulk 15**  **(hybrid*)*** | **G1 Bulk 15**  **(hybrid)** |
| **33** | **2.20** | **133.0137** | **[M-H]-** | **C_4_H_5_O_5_** | **0** | **115** | **Malic acid** | **+** | **+** | **+** | **+** | **+** | **+** | **+** | **+** | **+** | **+** |
| **34** | **9.40** | **175.0605** | **[M-H]-** | **C_7_H_11_O_5_** | **-0.857** | **157, 113, 85** | **Isopropyl malic acid isomer I** | **+** | **+** | **+** | **-** | **-** | **-** | **-** | **+** | **-** | **-** |
| **35** | **11.50** | **175.0606** | **[M-H]-** | **C_7_H_11_O_5_** | **-0,286** | **157, 113, 85** | **Isopropyl malic acid isomer II** | **+** | **+** | **+** | **+** | **+** | **+** | **+** | **+** | **+** | **+** |
| **36** | **2.95** | **191.0191** | **[M-H]-** | **C_6_H_7_O_7_** | **-0.419** | **173, 155, 111** | **Quinic acid** | **+** | **+** | **+** | **+** | **+** | **+** | **+** | **+** | **+** | **+** |
| **37** | **19.55** | **475.0875** | **-** | **-** | **-** | **299, 175, 157** | **Isopropyl malic acid derivative** | **+** | **+** | **+** | **+** | **+** | **-** | **+** | **+** | **+** | **+** |
|  | | | | | | | | | | | | | | | | | |
|  | | | | | | | | | | | | | | | | | |
| **Flavan-3-ols** | | | | | | | | | | | | | | | | | |
| **N°** | **tr**  **(min)** | **Exp.**  ***(m/z)*** | **Adduct** | **Formula Molecular**  **(Adduct)** | **Mass error (ppm)** | **Main fragments**  ***(m/z)*** | **Identification** | **INIA E-Tanin**  **(*L. uliginosus)*** | **INIA Gemma**  **(*L. uliginosus)*** | **Grasslands Maku**  **(*L. uliginosus)*** | **San Gabriel**  **(*L.corniculatus)*** | **INIA Rigel**  **(*L.corniculatus)*** | **LE 304-C1 Bulk 14**  **(*L.corniculatus)*** | **LE 304**  **(*L. corniculatus)*** | **INIA Draco**  **(*L. corniculatus)*** | **G5 Bulk 15**  **(hybrid*)*** | **G1 Bulk 15**  **(hybrid)** |
| **38** | **12.88** | **289.0712** | **[M-H]-** | **C_15_H_13_O_6_** | **-0.052** | **271, 245, 231, 205, 179, 165, 137, 109** | **Catechin** | **+** | **+** | **+** | **+** | **+** | **+** | **+** | **+** | **+** | **+** |
| **39** | **14.32** | **289.0712** | **[M-H]-** | **C_15_H_13_O_6_** | **-0.052** | **271, 245, 231, 205, 179, 165, 137, 109** | **Epicatechin** | **+** | **+** | **+** | **+** | **+** | **+** | **+** | **+** | **+** | **+** |
| **40** | **10.10** | **305.0659** | **[M-H]-** | **C_15_H_13_O_7_** | **-0.754** | **287, 261, 247, 221, 179, 165, 137, 125** | **Gallocatechin** | **+** | **+** | **+** | **+** | **+** | **-** | **-** | **-** | **-** | **+** |
| **41** | **12.04** | **305.0659** | **[M-H]-** | **C_15_H_13_O_7_** | **-0.754** | **287, 261, 247, 221, 179, 165, 137, 125** | **Epigallocatechin** | **+** | **+** | **+** | **+** | **+** | **+** | **+** | **+** | **+** | **+** |
| **42** | **10.38** | **451.1238** | **[M-H]-** | **C_21_H_23_O_11_** | **-0.532** | **289** | **(Epi) Catechin hexoside** | **+** | **+** | **+** | **-** | **+** | **-** | **+** | **-** | **-** | **+** |
| **Flavonols** | | | | | | | | | | | | | | | | | |
| **N°** | **tr**  **(min)** | **Exp.**  ***(m/z)*** | **Adduct** | **Formula Molecular**  **(Adduct)** | **Mass error (ppm)** | **Main fragments**  ***(m/z)*** | **Identification** | **INIA E-Tanin**  **(*L. uliginosus)*** | **INIA Gemma**  **(*L. uliginosus)*** | **Grasslands Maku**  **(*L. uliginosus)*** | **San Gabriel**  **(*L. corniculatus)*** | **INIA Rigel**  **(*L. corniculatus)*** | **LE 304-C1 Bulk 14**  **(*L. corniculatus)*** | **LE 304**  **(*L. corniculatus)*** | **INIA Draco**  **(*L. corniculatus)*** | **G5 Bulk 15**  **(hybrid*)*** | **G1 Bulk 15**  **(hybrid)** |
| **43** | **24.20** | **285.0397** | **[M-H]-** | **C_15_H_9_O_6_** | **-0.754** | **257, 243, 241, 213, 151, 107** | **Kaempferol** | **+** | **+** | **+** | **+** | **+** | **+** | **+** | **+** | **+** | **+** |
| **44** | **9.80** | **317.0290** | **[M-H]-** | **C_15_H_9_O_8_** | **-2.350** | **299, 271, 255, 231, 179, 151** | **Myricetin** | **+** | **+** | **+** | **+** | **+** | **+** | **+** | **+** | **+** | **+** |
| **45** | **18.80** | **477.1028** | **[M-H]-** | **C_22_H_21_O_12_** | **-1.058** | **357, 315, 300, 151** | **Isorhamnetin-hexoside** | **+** | **+** | **+** | **+** | **+** | **+** | **+** | **+** | **+** | **+** |
| **46** | **19.44** | **417.0820** | **[M-H]-** | **C_20_H_17_O_10_** | **-0,420** | **371, 327, 285, 257, 243, 179, 151** | **Kaempferol-pentoside** | **+** | **+** | **+** | **+** | **+** | **+** | **+** | **+** | **+** | **+** |
| **47** | **23.15** | **431.0973** | **[M-H]-** | **C_21_H_19_O_10_** | **-1,218** | **285, 255, 243, 179, 151** | **Kaempferol-deoxyhexoside** | **+** | **+** | **+** | **+** | **+** | **+** | **+** | **+** | **+** | **+** |
| **48** | **18.50** | **433.0765** | **[M-H]-** | **C_20_H_17_O_11_** | **-1,362** | **343, 301, 257, 179, 151** | **Quercetin-pentoside** | **+** | **+** | **+** | **+** | **+** | **+** | **+** | **+** | **+** | **+** |
| **49** | **18.68** | **447.0930** | **[M-H]-** | **C_21_H_19_O_11_** | **0.582** | **327, 285, 255, 179, 151** | **Kaempferol-hexoside** | **+** | **+** | **+** | **+** | **+** | **+** | **+** | **+** | **+** | **+** |
| **50** | **22.00** | **447.0924** | **[M-H]-** | **C_21_H_19_O_11_** | **-0,760** | **301** | **Quercetin-deoxyhexoside** | **+** | **+** | **+** | **+** | **+** | **+** | **+** | **+** | **+** | **+** |
| **51** | **17.49** | **463.0867** | **[M-H]-** | **C_21_H_19_O_12_** | **-2.062** | **343, 301, 271, 179, 151** | **Quercetin-hexoside** | **+** | **+** | **+** | **+** | **+** | **+** | **+** | **+** | **+** | **+** |
| **52** | **19.99** | **475.1240** | **-** | **-** | **-** | **433, 415, 343, 301, 271, 267, 179, 151** | **Quercetin derivative** | **+** | **+** | **+** | **-** | **-** | **-** | **-** | **-** | **-** | **-** |
| **53** | **16.00** | **479,0824** | **[M-H]-** | **C_21_H_19_O_13_** | **-0,355** | **317** | **Myricetin-hexoside** | **+** | **+** | **+** | **+** | **+** | **+** | **-** | **-** | **+** | **-** |
| **54** | **22.59** | **505.0972** | **[M-H]-** | **C_23_H_21_O_13_** | **-2,019** | **445, 301** | **Quercetin-acetylhexoside** | **+** | **+** | **+** | **+** | **+** | **+** | **+** | **+** | **+** | **+** |
| **55** | **16.43** | **563.1399** | **[M-H]-** | **C_26_H_27_O_14_** | **-0,329** | **431, 417, 285** | **Kaempferol-pentosyl-deoxyhexoside** | **+** | **+** | **+** | **+** | **+** | **+** | **+** | **+** | **+** | **+** |
| **56** | **17.38** | **577.1550** | **[M-H]-** | **C_27_H_29_O_14_** | **-1.273** | **431, 285** | **Kaempferol-deoxyhexosyl-deoxyhexoside** | **+** | **+** | **+** | **+** | **+** | **+** | **+** | **+** | **+** | **+** |
| **57** | **15.72** | **579.1341** | **[M-H]-** | **C_26_H_27_O_15_** | **-1,554** | **447, 433, 301** | **Quercetin-pentosyl-deoxyhexoside** | **+** | **+** | **+** | **+** | **+** | **+** | **+** | **+** | **+** | **+** |
| **58** | **16.20** | **593.1516** | **[M-H]-** | **C_27_H_29_O_15_** | **-0,253** | **447, 301** | **Quercetin-deoxyhexosyl-deoxyhexoside** | **+** | **+** | **+** | **+** | **+** | **+** | **+** | **+** | **+** | **+** |
| **59** | **13.52** | **595.1295** | **[M-H]-** | **C_26_H_27_O_16_** | **-0,697** | **577, 463, 433, 301** | **Quercetin-pentosyl-hexoside** | **+** | **+** | **+** | **+** | **+** | **+** | **-** | **-** | **-** | **-** |
| **60** | **15.22** | **609.1466** | **[M-H]-** | **C_27_H_29_O_16_** | **1.699** | **447, 463, 301** | **Quercetin-hexosyl-deoxyhexoside** | **+** | **+** | **+** | **+** | **+** | **+** | **+** | **+** | **+** | **+** |
| **61** | **16.40** | **609.1450** | **[M-H]-** | **C_27_H_29_O_16_** | **-0.928** | **447, 285** | **Kaempferol-hexosyl-hexoside** | **+** | **+** | **+** | **+** | **+** | **+** | **+** | **+** | **-** | **-** |
| **62** | **19.10** | **619.1660** | **[M-H]-** | **C_29_H_31_O_15_** | **-0,485** | **473, 431, 285** | **Kaempferol-acetyldeoxyhexosyl-deoxyhexoside** | **+** | **+** | **+** | **+** | **+** | **+** | **+** | **+** | **+** | **+** |
| **63** | **15.50** | **625.1403** | **[M-H]-** | **C_27_H_29_O_17_** | **-0,288** | **463, 301** | **Quercetin-hexosyl-hexoside** | **+** | **+** | **+** | **+** | **+** | **-** | **+** | **+** | **+** | **-** |
| **64** | **16.97** | **635.1602** | **[M-H]-** | **C_29_H_31_O_16_** | **-1,598** | **593, 559, 489, 431, 285** | **Kaempferol-deoxyhexosyl-acetylhexoside.** | **+** | **+** | **+** | **+** | **+** | **+** | **+** | **-** | **+** | **+** |
| **65** | **18.00** | **635.1608** | **[M-H]-** | **C_29_H_31_O_16_** | **-0,653** | **593, 557, 489, 447, 301** | **Quercetin-acetildeoxyhexosyl-deoxyhexoside** | **-** | **-** | **+** | **+** | **+** | **+** | **+** | **+** | **-** | **+** |
| **66** | **17.96** | **679.1511** | **-** | **-** | **-** | **647, 635, 619, 517, 335, 317, 301, 267** | **Quercetin glycoside derivative** | **+** | **+** | **+** | **+** | **+** | **+** | **+** | **+** | **+** | **+** |
| **67** | **15.86** | **695.1456** | **-** | **-** | **-** | **651, 549, 285** | **Kaempferol glycoside derivative** | **+** | **+** | **+** | **+** | **+** | **+** | **+** | **+** | **+** | **+** |
| **68** | **15.90** | **709.1971** | **[M-H]-** | **C_32_H_37_O_18_** | **-1,262** | **563, 431, 285** | **Kaempferol-deoxyhexosyl-pentosyl-deoxyhexoside** | **+** | **-** | **+** | **+** | **+** | **+** | **+** | **+** | **+** | **+** |
| **69** | **15.03** | **725.1910** | **[M-H]-** | **C_32_H_37_O_19_** | **-2.634** | **579, 563, 417, 285** | **Kaempferol-deoxyhexosyl-hexosyl-pentoside** | **+** | **+** | **+** | **+** | **+** | **+** | **+** | **+** | **+** | **+** |
| **70** | **14.49** | **739.2071** | **[M-H]-** | **C_33_H_39_O_19_** | **-1.975** | **593, 431, 285** | **Kaempferol-deoxyhexosyl-hexosyl-deoxyhexoside** | **+** | **+** | **+** | **+** | **+** | **+** | **+** | **+** | **+** | **+** |
| **71** | **14.25** | **741.1880** | **[M-H]-** | **C_32_H_37_O_20_** | **0.236** | **595, 463, 301** | **Quercetin-pentosyl-hexosyl-deoxyhexoside** | **+** | **+** | **+** | **+** | **+** | **+** | **+** | **+** | **+** | **+** |
| **72** | **15.42** | **755.2027** | **[M-H]-** | **C_33_H_39_O_20_** | **-1,026** | **609, 447, 301** | **Quercetin-deoxyhexosyl-hexosyl-deoxyhexoside** | **+** | **+** | **+** | **+** | **+** | **+** | **+** | **+** | **+** | **+** |
| **73** | **14.92** | **755.2038** | **[M-H]-** | **C_33_H_39_O_20_** | **0,430** | **609, 447, 285** | **Kaempferol, hexosyl-deoxyhexosyl-hexoside** | **+** | **+** | **+** | **+** | **+** | **+** | **+** | **+** | **+** | **+** |
| **74** | **13.49** | **771.1986** | **[M-H]-** | **C_33_H_39_O_21_** | **0.272** | **625, 463, 301** | **Quercetin-dihexosyl -deoxyhexoside** | **+** | **+** | **+** | **+** | **+** | **+** | **+** | **+** | **+** | **+** |
| **75** | **11.73** | **771.1981** | **[M-H]-** | **C_33_H_39_O_21_** | **-0,376** | **609, 447, 285** | **Kaempferol- dihexosyl-hexoside** | **+** | **+** | **+** | **-** | **-** | **-** | **-** | **-** | **-** | **+** |
| **76** | **16.08** | **809.2133** | **-** | **-** | **-** | **663, 609, 591, 485, 447, 285** | **Kaempferol glycoside derivative** | **+** | **+** | **+** | **+** | **+** | **-** | **-** | **-** | **-** | **+** |
| **77** | **15.36** | **825.2102** | **-** | **-** | **-** | **753, 679, 607, 446, 301** | **Quercetin glycoside derivative** | **+** | **+** | **+** | **-** | **-** | **-** | **-** | **-** | **-** | **+** |
| **78** | **15.24** | **839.5000** | **-** | **-** | **-** | **755, 693, 651, 609, 431, 285** | **Kaempferol glycoside derivative** | **-** | **+** | **+** | **-** | **-** | **-** | **-** | **-** | **-** | **-** |
| **79** | **18.20** | **839.2597** | **-** | **-** | **-** | **693, 609, 591, 285** | **Kaempferol glycoside derivative** | **+** | **+** | **+** | **+** | **+** | **+** | **+** | **+** | **-** | **+** |
| **80** | **14.83** | **857.2340** | **-** | **-** | **-** | **579, 285** | **Kaempferol glycoside derivative** | **+** | **-** | **-** | **-** | **-** | **-** | **-** | **-** | **-** | **-** |
| **81** | **14.42** | **857.2344** | **-** | **-** | **-** | **839, 813, 753, 711, 693, 667, 625, 607, 463, 446, 301** | **Quercetin glycoside derivative** | **-** | **+** | **+** | **-** | **-** | **-** | **-** | **-** | **-** | **-** |
| **82** | **12.99** | **865.1962** | **-** | **-** | **-** | **625, 463, 301** | **Quercetin glycoside derivative** | **-** | **-** | **-** | **+** | **+** | **+** | **+** | **+** | **+** | **-** |
| **83** | **20.86** | **875.2595** | **-** | **-** | **-** | **857, 815, 713, 695, 651, 625, 463, 301** | **Quercetin glycoside derivative** | **-** | **-** | **-** | **+** | **+** | **+** | **+** | **+** | **-** | **-** |
| **84** | **16.81** | **901.2402** | **-** | **-** | **-** | **755, 609, 285** | **Kaempferol glycoside derivative** | **+** | **+** | **+** | **+** | **+** | **-** | **-** | **-** | **-** | **+** |
| **85** | **15.58** | **917.2392** | **-** | **-** | **-** | **771, 755, 609, 285** | **Kaempferol glycoside derivative** | **+** | **+** | **+** | **-** | **-** | **-** | **-** | **-** | **-** | **-** |
| **86** | **16.71** | **931.2503** | **-** | **-** | **-** | **785, 609,285** | **Kaempferol glycoside derivative** | **+** | **+** | **+** | **+** | **+** | **-** | **+** | **-** | **-** | **+** |
| **87** | **15.72** | **941.3013** | **-** | **-** | **-** | **809, 795, 663, 609, 591, 447, 431, 285** | **Kaempferol glycoside derivative** | **-** | **+** | **+** | **-** | **-** | **-** | **-** | **-** | **-** | **-** |
| **88** | **16.10** | **947.2324** | **[M-H]-** | **C_39_H_47_O_27_** | **2.027** | **801, 771, 625, 607, 447, 301** | **Quercetin-glucurosyl-di hexosyl-deoxihexoside** | **+** | **+** | **+** | **-** | **-** | **-** | **-** | **-** | **-** | **+** |
| **Pranthocyanidins** | | | | | | | | | | | | | | | | | |
| **N°** | **tr**  **(min)** | **Exp.**  ***(m/z)*** | **Adduct** | **Formula Molecular**  **(Adduct)** | **Mass error (ppm)** | **Main fragments**  ***(m/z)*** | **Identification** | **INIA E-Tanin**  **(*L. uliginosus)*** | **INIA Gemma**  **(*L. uliginosus)*** | **Grasslands Maku**  **(*L. uliginosus)*** | **San Gabriel**  **(*L.corniculatus)*** | **INIA Rigel**  **(*L.corniculatus)*** | **LE 304-C1 Bulk 14**  **(*L.corniculatus)*** | **LE 304**  **(*L.corniculatus)*** | **INIA Draco**  **(*L. corniculatus)*** | **G5 Bulk 15**  **(hybrid*)*** | **G1 Bulk 15**  **(hybrid)** |
| **89** | **12.00** | **577.1341** | **[M-H]-** | **C_30_H_25_O_12_** | **-0.875** | **559, 451, 425, 407, 289** | **(E)-Cat-(E)-Cat** | **+** | **+** | **+** | **+** | **+** | **+** | **+** | **+** | **+** | **+** |
| **90** | **12.54** | **577.1341** | **[M-H]-** | **C_30_H_25_O_12_** | **-0.875** | **559, 451, 425, 407, 289** | **(E)-Cat-(E)-Cat** | **+** | **+** | **+** | **+** | **+** | **+** | **+** | **+** | **+** | **+** |
| **91** | **9.63** | **593.1287** | **[M-H]-** | **C_30_H_25_O_13_** | **-1,382** | **575, 467, 441, 425, 107, 303, 289** | **(E)-Cat-(E)-Gall** | **+** | **+** | **+** | **+** | **+** | **+** | **+** | **+** | **+** | **+** |
| **92** | **10.74** | **593.1287** | **[M-H]-** | **C_30_H_25_O_13_** | **-1,382** | **575, 467, 441, 425, 107, 303, 289** | **(E)-Cat-(E)-Gall** | **+** | **+** | **+** | **-** | **-** | **-** | **-** | **-** | **-** | **+** |
| **93** | **11.30** | **593.1287** | **[M-H]-** | **C_30_H_25_O_13_** | **-1,382** | **575, 467, 441, 425, 107, 303, 289** | **(E)-Cat-(E)-Gall** | **+** | **+** | **+** | **+** | **+** | **+** | **+** | **+** | **+** | **+** |
| **94** | **12.40** | **593.1287** | **[M-H]-** | **C_30_H_25_O_13_** | **-1,382** | **575, 467, 441, 425, 107, 303, 289** | **(E)-Cat-(E)-Gall** | **+** | **+** | **+** | **-** | **-** | **-** | **-** | **-** | **-** | **+** |
| **95** | **7.44** | **609.1250** | **[M-H]-** | **C_30_H_25_O_14_** | **0.928** | **591, 515, 441, 423, 305** | **(E)-Gall-(E)-Gall** | **+** | **+** | **+** | **-** | **-** | **-** | **-** | **-** | **-** | **+** |
| **96** | **9.00** | **609.1250** | **[M-H]-** | **C_30_H_25_O_14_** | **0.928** | **591, 515, 441, 423, 305** | **(E)-Gall-(E)-Gall** | **+** | **+** | **+** | **-** | **-** | **-** | **-** | **-** | **-** | **+** |
| **97** | **9.58** | **609.1250** | **[M-H]-** | **C_30_H_25_O_14_** | **0.928** | **591, 515, 441, 423, 305** | **(E)-Gall-(E)-Gall** | **+** | **+** | **+** | **-** | **-** | **-** | **-** | **-** | **-** | **+** |
| **98** | **9.08** | **865.1972** | **[M-H]-** | **C_45_H_37_O_18_** | **-0.919** | **847, 755, 739, 713, 695, 577, 543, 451, 407, 363, 289, 287** | **(E)-Cat-(E)-Cat-(E)-Cat** | **+** | **+** | **-** | **+** | **+** | **+** | **+** | **+** | **-** | **+** |
| **99** | **11.80** | **881.1935** | **[M-H]-** | **C_45_H_37_O_19_** | **0.670** | **863, 755, 729, 711, 695, 593, 423, 407, 289** | **(E)-Cat-(E)-Gall-(E)-Cat** | **+** | **+** | **+** | **+** | **+** | **+** | **-** | **-** | **-** | **+** |
| **100** | **10.30** | **897.1866** | **[M-H]-** | **C_45_H_37_O_20_** | **-1,365** | **837, 771, 729, 711, 607, 577, 579, 439, 385, 305** | **(E)-Cat-(E)-Gall-(E)-Gall** | **+** | **+** | **+** | **-** | **-** | **-** | **-** | **-** | **-** | **+** |
| **101** | **10.51** | **897.1866** | **[M-H]-** | **C_45_H_37_O_20_** | **-1,365** | **837, 771, 729, 711, 607, 577, 579, 439, 385, 305** | **(E)-Cat-(E)-Gall-(E)-Gall** | **+** | **+** | **+** | **-** | **-** | **-** | **-** | **-** | **-** | **+** |
| **102** | **11.08** | **897.1866** | **[M-H]-** | **C_45_H_37_O_20_** | **-1,365** | **837, 771, 729, 711, 607, 577, 579, 439, 385, 305** | **(E)-Cat-(E)-Gall-(E)-Gall** | **+** | **+** | **+** | **-** | **-** | **-** | **-** | **-** | **-** | **+** |
| **103** | **12.20** | **897.1866** | **[M-H]-** | **C_45_H_37_O_20_** | **-1.365** | **837, 771, 729, 711, 607, 577, 579, 439, 385, 305** | **(E)-Cat-(E)-Gall-(E)-Gall** | **+** | **+** | **+** | **-** | **-** | **-** | **-** | **-** | **-** | **+** |
| **104** | **9.26** | **913.1818** | **[M-H]-** | **C_45_H_37_O_21_** | **-1,029** | **895, 787, 745, 727, 609, 483, 441, 423, 287, 289, 303, 305** | **(E)-Gall-(E)-Gall(E)-Gall** | **+** | **+** | **+** | **-** | **-** | **-** | **-** | **-** | **-** | **-** |
| **105** | **10.09** | **913.1818** | **[M-H]-** | **C_45_H_37_O_21_** | **-1,029** | **895, 787, 745, 727, 609, 483, 441, 423, 287, 289, 303, 305** | **(E)-Gall-(E)-Gall(E)-Gall** | **+** | **+** | **+** | **-** | **-** | **-** | **-** | **-** | **-** | **+** |

(E)Cat: catechin or epicatechin; (E)Gall: gallocatechin or epigallocatechin
